# A comprehensive SARS-CoV-2 and COVID-19 review, Part 1: Intracellular overdrive for SARS-CoV-2 infection

**DOI:** 10.1038/s41431-022-01108-8

**Published:** 2022-05-16

**Authors:** David A. Jamison, S. Anand Narayanan, Nídia S. Trovão, Joseph W. Guarnieri, Michael J. Topper, Pedro M. Moraes-Vieira, Viktorija Zaksas, Keshav K. Singh, Eve Syrkin Wurtele, Afshin Beheshti

**Affiliations:** 1 COVID-19 International Research Team, Medford, MA USA; 2 Department of Nutrition & Integrative Physiology, Florida State University, Tallahassee, FL USA; 3 Fogarty International Center, National Institutes of Health, Bethesda, MD USA; 4 Center for Mitochondrial and Epigenomic Medicine, Children’s Hospital of Philadelphia, Philadelphia, PA USA; 5 Department of Oncology, The Sidney Kimmel Comprehensive Cancer Center at Johns Hopkins, Baltimore, MD USA; 6 Department of Genetics, Evolution, Microbiology and Immunology, Institute of Biology, University of Campinas, Campinas, SP Brazil; 7 Obesity and Comorbidities research Center (OCRC), University of Campinas, Campinas, SP Brazil; 8 Experimental Medicine Research Cluster, University of Campinas, Campinas, Brazil; 9 Center for Translational Data Science, University of Chicago, Chicago, IL USA; 10 Department of Genetics, Heersink School of Medicine, University of Alabama at Birmingham, Birmingham, AL USA; 11 Center for Metabolic Biology, Bioinformatics and Computational Biology, and Genetics Development, and Cell Biology, Iowa State University, Ames, IA USA; 12 Stanley Center for Psychiatric Research, Broad Institute of MIT and Harvard, Cambridge, MA USA; 13 KBR, Space Biosciences Division, NASA Ames Research Center, Moffett Field, CA USA

**Keywords:** Viral infection, Infection

## Abstract

COVID-19, the disease caused by SARS-CoV-2, has claimed approximately 5 million lives and 257 million cases reported globally. This virus and disease have significantly affected people worldwide, whether directly and/or indirectly, with a virulent pathogen that continues to evolve as we race to learn how to prevent, control, or cure COVID-19. The focus of this review is on the SARS-CoV-2 virus’ mechanism of infection and its proclivity at adapting and restructuring the intracellular environment to support viral replication. We highlight current knowledge and how scientific communities with expertize in viral, cellular, and clinical biology have contributed to increase our understanding of SARS-CoV-2, and how these findings may help explain the widely varied clinical observations of COVID-19 patients.

## Introduction

As of November 21st, 2021, over 257 million cases of coronavirus disease 2019 (COVID-19) have been reported, and more than 5 million lives claimed globally [1]. The disease is caused by the severe acute respiratory syndrome coronavirus 2 (SARS-CoV-2). Development of vaccines against SARS-CoV-2 provide a major step forward in reducing COVID-19’s impact. However, the pandemic is ongoing, and the continued viral transmission allows for accumulation of mutations in the viral genome, which can provide advantages in replication, immune escape, increased transmissibility, or diagnostic detection failure [2]. With the quickly evolving SARS-CoV-2 variants and the slow rate of vaccination globally, it is critical to fully understand this novel virus and disease.

**Fig. 1:**
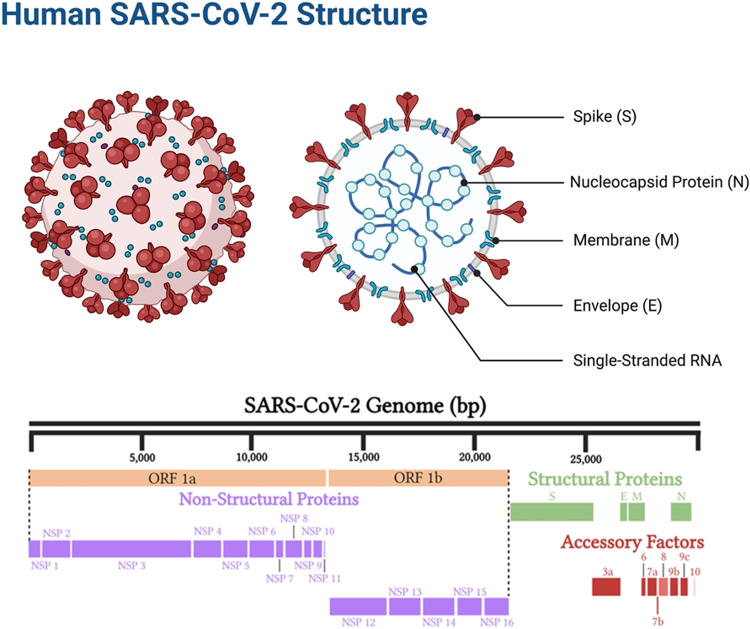
SARS-CoV-2 structure. Structural elements of the virus, including the spike protein, envelope, membrane, and internal components such as the viral single-stranded RNA and nucleocapsid proteins (above). SARS-CoV-2 genome components (below).

Coronaviruses are named as such because the S proteins resemble a halo or corona on scanning electron microscope imagery [3]. SARS-CoV-2 belongs to the genus Betacoronavirus. Of the human Betacoronavirus, including OC43, HKU1, SARS-CoV-1, and Middle East Respiratory Syndrome-Coronavirus (MERS-CoV) [4]. SARS-CoV-2 bears the highest genetic sequence similarity to SARS-CoV-1 [5]. Accordingly, COVID-19, caused by SARS-CoV-2, resembles SARS, caused by SARS-CoV-1, in many ways, but with some important differences [6]. Key characteristics of SARS-CoV-1 and 2 include: 1) a positive-sense RNA virus with a large genome of ~30 kilobases; 2) a large, enveloped virus containing a helical nucleocapsid with the virus’s genetic code, with an exterior studded in several spike proteins that facilitate the infection of host cells), and 3) similar genomic structures. The first 2/3 of both genomes encodes for two macro polypeptides pp1a/pp1b (see Fig. 1). Pp1a/pp1b are auto-proteolytically processed to generate 16 non-structural proteins (NSP).

The main virus-specific functions of the SARS-CoV-2 NSPs are: NSP1 - cellular mRNA degradation, global translation inhibition; NSP2 - cell cycle progression disruption; NSP3 - formation of double-membrane vesicles (DMVs; SARS-CoV-2 protease); NSP4 - formation of DMVs; NSP5 - main SARS-CoV-2 protease; NSP6 - formation of DMVs, NSP7 - replication complex; NSP8 – primase; NSP9 - RNA binding protein; NSP10 - cofactor of NSP14 & NSP16; NSP11 - unknown, NSP12 - RNA-dependent RNA polymerase; NSP13 - RNA helicase, 5ʹ phosphatase, NSP14 - N7-MTase, 3ʹ-5ʹ exonuclease; NSP15 – endonuclease; and NSP16–2ʹ-O-MTase, mRNA capping.

**Fig. 2:**
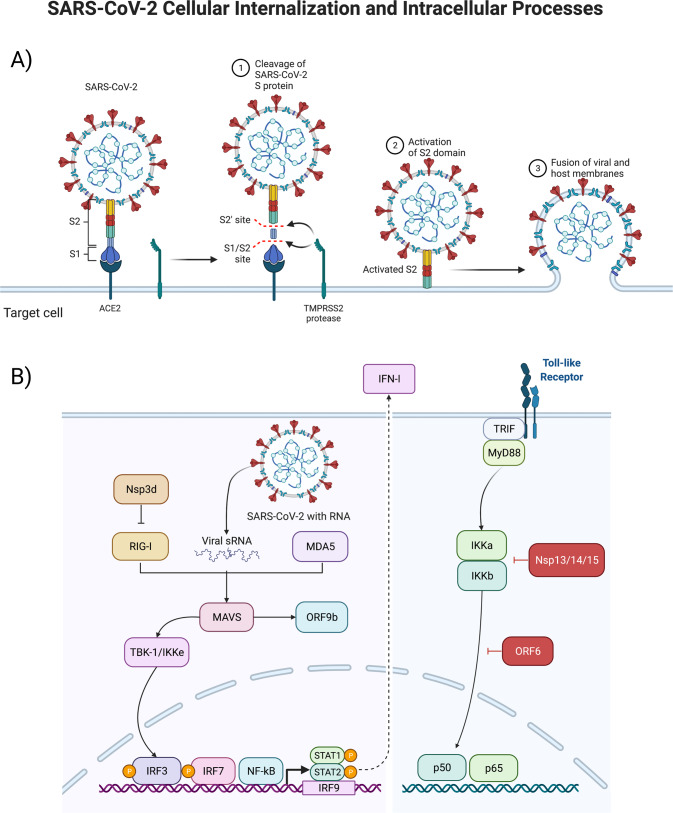
SARS-CoV-2 binding, intracellular internalization, and intracellular processes. Structural interactions between the virus and target cell, including the viral spike protein, ACE2-receptor, TMPRSS2 reaction to cleave and begin the viral intracellular internalization (above, **A**), and consequent signal transduction pathways stimulated by the virus as it hijacks pathways to turn the infected cell into a SARS-CoV-2 producing factory (below, **B**).

The remaining 1/3 of the SARS-CoV-2 genome encodes for the structural proteins S (spike), E (envelope), M (membrane), and N (nucleocapsid), and several open reading frames (ORFs; (3a, 6, 7a, 7b, 8, 9b, and 10) [7]. The S protein binds the host cell receptor, which for SARS-CoV-1/2 is the human angiotensin-converting enzyme 2 (hACE2) (see Fig. 2 and Supplementary Table 1). These proteins share homology and function with SARS-CoV-1.

There are two notable differences between SARS-CoV-1 and SARS-CoV-2. First is the presence of the ORF8 polypeptide found in SARS-CoV-2 but not in SARS-CoV-1. SARS-CoV-1 has a 29 nucleotide (nt) deletion (del) which splits it into ORF8a and ORF8b. Second, SARS-CoV-2 contains a gene encoding a novel orphan protein, ORF10, which is not present in SARS-CoV-1 [7].

SARS-CoV-2’s evolutionary rate has been estimated to be around 9’×’10^−4^ substitutions per site per year [8], while also having a high transmissibility, large portion of asymptomatic cases [9], large pool of susceptible hosts to replicate in [10, 11], and on-going environmental pressures (e.g., low vaccination rates and changes in policies allowing human carriers to continue to transmit the virus), which have allowed SARS-CoV-2 to accumulate mutation in its genome.

Mutations have been detected in all parts of the viral genome, including in the leader 5ʹ untranslated region (UTR), orf1ab (NSP1, NSP2, NSP3, NSP6, NSP12, NSP13, and NSP14), spike, ORF3a, ORF8, nucleocapsid, and ORF10 [8]. These genomic changes have been shown to influence viral immune evasion, inflammasome interaction, helicase, exonuclease proofreading mechanism, the activity of the RNA-dependent RNA polymerase (RdRp) and thereby viral replication, infectivity, and cell release [12].

Mutations associated with the spike are of particular interest, as they influence human-to-human transmission, as well as human-to-animal passage. Within the spike, mutations tend to fall into four general classes, those that affect the receptor-binding domain (RBD), which are of importance because some may provide both immune escape or a fitness advantage, as well as facilitate reverse zoonotic events. There are some mutations that occur in the N-terminal domain (NTD), which is the portion most exposed on the virus surface. There is evidence for immune selection in this region, and preliminary evidence that at least one of these changes (delH69/delV70) could improve fitness [13]. Mutations in or near the furin cleavage site, and several groupings close to the D614G mutation, possibly affect infection efficiency and can also be important for neutralizing antibodies.

This large SARS-CoV-2 genome diversity has been categorized by different nomenclature systems, describing variants of varied public health interest or concern. Pango lineages B.1.1.7 (Alpha), B.1.351 (Beta), P.1 (Gamma) and B.1.617.2 (Delta) have been classified as “variants of concern” (VOC) because they present mutations that have been shown to impact diagnostics, treatments, or vaccines, conferring increased transmissibility and increased disease severity. The impact of these mutations highlights the need for further research not only on the mechanisms of SARS-CoV-2 intracellular processes, but also how the extracellular environment may lead to further spread of the virus and subsequent public health burden.

The SARS-CoV-2 virus can exert physiological effects by directly infecting cells and via intercellular signaling by the infected cells. In this review, we provide insight into SARS-CoV-2 infection and intracellular host responses (targets, pathways, networks, biological processes, and functional adaptations) to viral invasion. We emphasize a canonical set of reactions induced by SARS-CoV-2, which we have organized for the reader’s consideration. However, there is tremendous variation in cellular responses to SARS-CoV-2, depending on factors including the cell type, organ type, metabolic and physiological context, patient genetics, individual clinical characteristics (e.g., age, sex, comorbidities), and stage/severity in the COVID-19 disease.

This is the first of a three-part comprehensive series of linked reviews on SARS-CoV-2 covering: intracellular effects (present study); extracellular consequences (review 2); and current and potential therapeutics (review 3). This review and the two that will follow aim to provide a foundational understanding of the current knowledge on SARS-CoV-2, from basic biology to clinical outcome and therapy avenues, that highlight future areas of research and could help inform public health interventions across the world.

### Infected tissue and cell types

SARS-CoV-2 targets the nasal cavity and lungs; however, the detailed cellular tropism remains unclear, and likely varies among individuals. Furthermore, there is increased variability of viral cellular tropism with the emergent SARS-CoV-2 variants, which include Alpha, Beta, Gamma, Epsilon, Eta, Iota, Kappa, 1.617.3, Mu, Zeta, and in particular, Delta and Omicron, as well as the various lineages of each variant [14]. This is related with SARS-CoV-2’s mutation ability affecting its antigenic phenotype to circumvent immunity. The spike protein mediates attachment of the virus to host cell-surface receptors and fusion between virus and cell membranes; it is also the principal target for neutralizing antibodies generated following infection, and is the component for both mRNA and adenovirus-based vaccines [15]. Several studies have contributed to the current understanding of how mutations in the SARS-CoV-2 spike protein affect neutralization and emergence of new strains, which include studies of traditional escape mutation, targeted characterization of particular mutations, and wider investigations of large numbers of circulating variants [16]. These are active areas of research, in particular given the continued emergence of new lineages of new variants. A study of human, bat, non-human primate, and mouse cell lines showed various cell types were susceptible to the virus. These included pulmonary, intestinal, hepatic, renal, and neuronal, with cell lines expressing the hACE-2 receptor (hACE-2) having a generally greater viral load [17]. Although cell lines do not reflect physiological conditions, this research indicates that SARS-CoV-2 can infect many cell types, and that hACE-2 provides a critical entry mechanism [18]. Epithelial, vascular endothelial, pancreas, and mucosal cell types can all be infected by the virus [19–21].

Several investigations have employed 3D organoid cultures to simulate more physiological conditions than cell cultures [22]. In one such study, lung and colonic organoid models showed SARS-CoV-2 infection was reduced when various SARS-CoV-2 entry inhibitors were applied [22]. Another study illustrated the flexibility of different organoid models, such as pancreatic endocrine cells, liver organoids, cardiomyocytes, and dopaminergic neurons from human pluripotent stem cells, and adult primary cells (human islets, hepatocyte, and cholangiocytes) to test viral effects such as cytokine production, gene expression, and other physiological responses. The resultant data correlated well with some patient autopsy samples [22] indicating organoids provide a valuable disease modeling tool [18].

In one study of post-mortem patients, immunohistochemistry and immunofluorescence revealed viral antigen (spike protein) in pneumocytes and hyperplastic cells around the bronchioles, mucosal epithelia, submucosal glands, gland ducts of the trachea, glands of the small intestine, distal tubules and collecting ducts of the kidneys, islets of Langerhans, glands and intra-islet ducts of the pancreas, and vascular tissues of the brain and heart [23]. Few viral antigens were present in the large intestine and renal proximal tubules, and none in the liver. A follow-up colocalization analysis showed ACE2 and viral antigen in the lung, trachea, small intestine, kidney, pancreas, and heart. In the brain, ACE2-expressing cells were detected, but they were negative for the viral antigen [23]. Endothelial cells of multiple organs were infected, supporting the clinical observations of endotheliitis in some COVID-19 patients.

Single-cell RNA sequencing (scRNA-seq) demonstrated ACE2 receptor expression was primarily restricted to lung pneumocytes, gut absorptive enterocytes, and nasal mucosa goblet secretory cells [24]. In general, the distribution of ACE2 receptors may in part explain the systemic diversity and range of SARS-CoV-2’s effects. Further research into infection of these cell types versus others in mucosal barrier organs will be important to determine cell-types that serve as initial entry ways for the virus into the body.

Human autopsy studies [21] have shown that SARS-CoV-2 infects multiple organs including lungs, pharynx, liver, nasal mucosa, trachea, intestines, skin, pancreas, kidney, brain, and heart. A study of 27 patients showed multi-organ tropisms (lung, pharynx, heart, liver, brain, and kidneys), with the highest levels of SARS-CoV-2 copies per cell, as detected by in situ hybridization and indirect immunofluorescence, in the respiratory tract, and lower levels in the kidneys, liver, heart, and brain [21]. Transcriptional profiling of nasopharyngeal swabs, patient autopsy, and body-wide tissues (e.g. heart, liver, lung, kidney, and lymph nodes), provided further evidence of the physiologically systemic effects of SARS-CoV-2 [24].

These studies suggest that the virus has a varying range of expression within each organ, which may be influenced by levels of the ACE2-receptor and related entry factors (Transmembrane protease, serine-2 [TMPRSS2], transferrin receptor protein 1 [TRFC1], cluster of differentiation 4 [CD4], and neuropilin-1 [NRP1]) within each organ-type [24]. This further highlights the varied organ and tissues that are capable of being infected by the virus, and the resultant wide-range of patient symptoms.

The physiological status of the individual significantly affects COVID-19 morbidity and mortality [25, 26]. Notably, patients with pre-existing conditions of obesity, hypertension, and diabetes have a less favorable disease outcome, likely in part due to the elevated levels of inflammation and metabolic disturbances associated with those conditions [25]. Conversely, SARS-CoV-2 infection may exacerbate pre-existing conditions, leading to more severe COVID-19 outcome [27].

### SARS-CoV-2 Receptors – Angiotensin Converting Enzyme-2 (ACE2)

The cellular surface receptor ACE2, a key regulator of the Renin-Angiotensin Aldosterone System (RAAS). It is speculated to be the primary SARS-CoV-2 viral target for entry. SARS-CoV-2 is thought to infect multiple organs in part due to the widespread distribution, expression, and polymorphisms of ACE2 [28, 29].

ACE2’s molecular function in the human RAAS pathway is to cleave Angiotensin I to produce Angiotensin 1–9, and break down Angiotensin II into Angiotensin 1–7. RAAS moderates blood pressure and osmolarity by means of hormonal feedback control. In response to binding of ACE2 to the ACE2 receptor (ACE2R), blood vessels vasoconstrict. This process is mediated by G-protein-signaling, activating phospholipase C and increasing cytosolic Ca^2+^ concentrations. ACE2 also plays an important role in inactivating Des-Arg9-Bradykinin (DABK), a bradykinin involved in inflammation. This inactivation promotes C-X-C motif chemokine 5 (CXCL5), macrophage inflammatory protein-2 (MIPS2), keratinocytes-derived chemokine (KC), and tumor necrosis factor-〈 (TNF-α) activity, drawing leukocytes into the affected tissues [30].

Decreased ACE2 receptor expression can have detrimental effects. Computational models of COVID-19 suggest the role of a bradykinin storm in the pathophysiology of the disease. In this model, the Kallikrein-Kinin and Renin-Angiotensin-Aldosterone Systems are integrated, with cross-talk mediated by the degradation of bradykinin by ACE and prolylcarboxypeptidase [31]. This behavior makes the SARS-CoV-2 spike protein behave akin to an ACE-inhibiting drug [32]. Thus, disruption of ACE2 expression from SARS-CoV-2 binding can lead to altered tissue function and exacerbate disease.

The ~600-kDa trimeric S proteins can bind to ACE2 through the RBD required for membrane fusion (see Fig. 2). The binding initiates viral internalization, with the cleavage of S1/S2 inducing a conformational change from prefusion into post-fusion. S1 consists of the NTD, the RBD, and subdomain 1 and 2 (SD1 and SD2). S2 contains the hydrophobic fusion peptide and is responsible for the viral and cell membrane fusion [33]. SARS-CoV-2 S-protein shows varying states of conformational shifts of the RBD site progressing towards proteolytic processing, making the viral RBD more accessible to ACE2, with the cleavage at the S1/S2 leading towards RBD open confirmation and viral internalization [33, 34]. The S- and RBD-viral sites are notable for affecting transmission and disease severity, and variants have been shown to accumulate mutations at these sites leading to increased S- and RBD affinity with ACE2 [35]. Understanding the biology of the SARS-CoV-2 surface interactions will help elucidate how the virus can invade multiple organ systems and cell types.

### Calcium Ion (Ca^2+^) Signaling

**Fig. 3:**
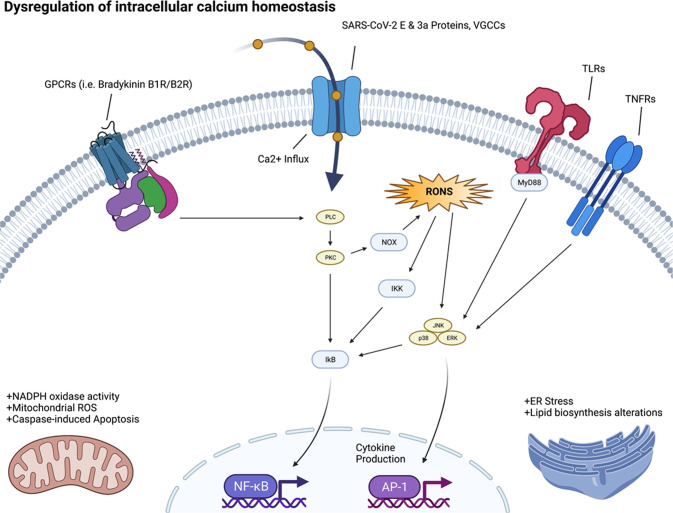
SARS-CoV-2 effects on Ca^2+^ signaling. Structural elements of the virus, including the spike protein, envelope, membrane, and internal components such as the viral single-stranded RNA and nucleocapsid proteins (above).

The calcium ion (Ca^2+^) is essential for many aspects of cellular physiology and viral replication. Experimental data on the relation between Ca^2+^ signaling and SARS-CoV-2 infection and replication is sparse. However, studies of other coronaviruses (e.g., SARS-CoV-1, MERS-CoV) have reported that these viruses utilize Ca^2+^ for host fusion [36]. The fusion protein (FP) of MERS-CoV binds to one Ca^2+^ ion, while the SARS-CoV-2 spike (S) protein has two FP domains, FP1 and FP2, and binds to two Ca^2+^ ions for host cell entry [37]. SARS-CoV-2 appears to affect cellular function by altering the host Ca^2+^ homeostasis in ways that promote viral infection and reproduction (see Fig. 3). One mechanism is through disruption of calcium channels and pumps (e.g., voltage-gated calcium channels (VGCCs), receptor-operated calcium channels, store-operated calcium channels, transient receptor-potential ion channels, and Ca^2+^-ATPase) [28, 37]. This leads to increased intracellular Ca^2+^ concentrations, resulting in virus-induced cell lysis [28, 37]. The interaction between the virus and VGCCs may also promote virus-host cell fusion for entry [28].

Viroporins, transmembrane pore-forming proteins that alter membrane permeability to ions including Ca^2+^ by forming membrane channels, are a characteristic of a diversity of virus. SARS-CoV-1/2 each encode viroporins. SARS-CoV-1 encodes for three viroporin proteins ORF3a, E and ORF8b, which alter ion homeostasis within the cell, and have important roles in pathogenesis and promoting viral fitness. SARS-CoV-2 encodes two of these viroporin proteins, E and ORF3a; however, the ORF8 protein of SARS-CoV-2 is highly divergent from SARS-CoV-1 ORF8b and lacks the viroporin sequence of SARS-CoV-1 ORF8b.

The E and ORF3a proteins of coronaviruses impact Ca^2+^ homeostasis in the host, by acting as calcium ion channels, enhancing the virion’s entry and replication potential [38]. The SARS-CoV-2-E protein is a 76 amino acid (aa) integral membrane protein with one transmembrane domain (TMD) that allows the E protein to form protein-lipid channels in membranes that promote permeability to Ca^2+^ ions. The SARS-CoV-2-ORF3a protein is 274 aa in length, harbors three helical TMD, and is a Na^+^ or Ca^2+^ ion channel protein. The alteration of Ca^2+^ homeostasis by SARS-COV-1-E and SARS-COV-2-E proteins promotes SARS-CoV-1/2 fitness and elicits the production of chemokines and cytokines, contributing to pathogenesis. Ion channel activity modulation by the SARS-CoV-1-ORF3a protein also modulates viral release [39]. Therefore, when SARS-CoV-2 infects the human body, the resultant dysregulation of Ca^2+^ homeostasis may contribute to morbidity and mortality. COVID-19 patients have been noted to have low serum calcium levels overall [40].

We speculate that Ca^2+^ dysregulation could lead to increased cellular oxidative stress and shifts in metabolic activity. Low Ca^2+^ may also be coupled with viral infection and internalization through the ACE2R, which synergizes with Ca^2+^ signaling pathways. Understanding these reverberations will increase our insight into the basic biology of the effects of SARS-CoV-2 infection on the various organ systems.

### Intracellular signaling

Viral infection and hijacking of cell-surface receptors begin to trigger activation of multiple intracellular pathways in addition to Ca^2+^ signaling. As infection proceeds, SARS-CoV-2 manipulates, or totally reprograms, the normal metabolism and signaling of the host cell, optimizing the molecular environment to enable the viral replication cycle. This involves interfering with signaling pathways that regulate processes of DNA repair and replication, immune response, transcription, metabolism, cell cycle, and apoptosis [39].

SARS-CoV-2 infection alters phosphoinositide 3-kinase (PI3K)/protein kinase B (AKT), Type I and III interferon, transforming growth factor-β (TGF-β), Toll-like receptors (TLR), and nuclear factor kappa-light chain enhancer (NF-κB) pathways. These pathways are dysregulated in the setting of SARS-CoV-2 to antagonize host antiviral responses and are vital for viral replication, entry, propagation, and/or apoptosis/viral release. For instance, severe COVID-19 is characterized by an inflammatory profile dominated by NF-κB activity [41]. The SARS-CoV-2-encoded NSP13 and Open Reading Frame 9c (ORF9c) proteins can interact directly with elements of the transducin-like enhancer (TLE) family of proteins and thus regulate the NF-κB inflammatory response [42]. While broad activation of NF-κB is induced by a variety SARS-CoV-2-encoded products, Open Reading Frame 7a (ORF7a) specifically is a potent stimulator of NF-κB associated proinflammatory chemo- and cytokines, which are elevated in the presence of severe COVID-19. NF-κB plays a similar role in other coronavirus infections.

Host antiviral immunity requires an optimal and coordinated response to control viral infections; this immunity is mediated by several host sensors, notably pattern recognition receptors (PRR). PRRs identify damage- and pathogen-associated molecular patterns (DAMPs and PAMPs, respectively). SARS-CoV-2 infects the cell via the endosomal compartment, and may activate TLRs, such as TLR4, resulting in increased NF-κB activity and expression [42, 43]. The MyD88-mediated TRIF activation of TLR downstream pathways triggers the nuclear translocation of NF-κB, IFN regulatory factor 3 (IRF-3), and IFN regulatory factor 7 (IRF-7), resulting in the expression of innate immunity proinflammatory cytokines (interleukin-1 [IL-1], interleukin-6 [IL- 6], TNF-α) and interferons (IFNs). Continuous activation of TLR can increase MyD88 and Interleukin-1β (IL-1β), which then can further activate NF-κB [43]. RNA viruses are detected by several sensors, such as TLRs 3, 7 and 8. TLR3 recognizes double stranded RNA, while TLR7 and TLR8 single stranded RNA. In addition to ssRNA and dsRNA, viral proteins can act as PAMPS and potentiate inflammatory signaling through the stimulation of surface TLRs. Interestingly, in SARS-CoV-2, TLR2 is a critical mediator of envelope protein detection and driver of pathogenesis through inflammatory process augmentation [44]. Some individuals with severe COVID-19 have mutations in genes associated with type I and III IFN pathways [45]. Ten percent of individuals that progress to severe COVID-19 pneumonia display elevated amounts of neutralizing antibodies against type I IFN-α2 and IFN-ω [46]; these antibodies are not present in healthy or asymptomatic individuals. Of note, albeit TLR3 activation is critical for viral clearance, TLR3 hyperactivation can lead to a cytokine storm and the subsequent severe COVID-19. Other receptors are also involved in SARS-CoV-2 recognition, such as the proteins of retinoic acid-inducible gene I (RIG-I) and melanoma differentiation-associated gene 5 (MDA-5). Once inside the cell, double strand viral RNA can be recognized by RIG-I/MDA5, thus initiating an antiviral response through mitochondrial antiviral signaling (MAVS). MAVS activated the downstream pathways, IκB kinase α/β (IKK) and TBK1/IKKε, leading to translocation of NF-κB and/or IRF3 into the nucleus and induction of genes involved with innate antiviral immunity and the subsequent induction of IFN-stimulated genes (ISGs).

Inhibition of IFNs and ISGs is a tactic used by several viruses to evade host antiviral responses [47], and SARS-CoV-2-mediated IFNs and ISGs dysregulation appears to be an important strategy used by this virus to replicate and disrupt immune homeostasis. Furthermore, therapies with type I and III IFNs alone or combined with other drugs suppressed SARS-CoV-2, ameliorating COVID-19 disease [48].

The cytokine TGF-β triggers the Janus kinases (JAKs) / signal transducer and activator of transcription (STAT) proteins (JAK/STAT) pathway in certain contexts, while suppressing it in others [49]. It has been proposed that SARS-CoV-2 proteins, particularly NSP1 and ORF6, may dysregulate STAT1 and STAT3, leading to a positive feedback loop where coagulopathy triggers TLR4 via PAI-1 binding, circularly activating STAT; for this reason, therapeutic targeting of the Janus kinase pathway has been proposed [50].

The innate immune response is a first step to protecting against pathogens, which stimulate the interferon signaling pathway and expression of IFN-I, leading to an antiviral cellular response [51]. Coronaviruses have developed mechanisms to hinder IFN-expression and reduce the production of IFN. This suppression has been shown to correlate with disease severity and mortality [52]. This holds true for SARS-CoV-2, with recent studies showing that viral proteins ORF6, ORF8, and nucleocapsid being potent inhibitors of the IFN-I signaling pathway [53].

### Metabolic adaptations

Viruses rely on host cell machinery to propagate, promoting anabolism for generation of macromolecules needed for virion replication and assembly (see Fig. 2). Consequently, viral proteins (see Supplementary Table 1) affect intracellular pathways, leading to subsequent adaptations by the cellular metabolism where the mitochondria plays a central role.

We reported recently through study of COVID-19 patient samples (i.e., nasopharyngeal swab samples, various organs from autopsy COVID-19 samples, murine lung tissues, and various organs from hamsters being infected with SARS-CoV-2) that heavy suppression occurs of mitochondrial functions in various organs [54]. Specifically, in the course of SARS-CoV-2 infection, the virus blocks the transcription of discrete groups of mitochondrial genes from major bioenergetic organs, while upregulation occurs in others as a compensatory mechanism to rescue the damage occurring in the major bioenergetic organs. This demonstrated a dynamic evolution of mitochondrial gene expression and cellular energetics as the virus progresses from one organ to the next. Transcriptomic changes in the nasopharyngeal infected samples revealed that during initial SARS-CoV-2 infection, nDNA coded mitochondrial genes are blocked and the co-inhibited genes were found to group together as components of preassembly modules of the mitochondrial oxidative phosphorylation (OXPHOS) complexes I, II, III, IV and V. At the time of death for COVID-19 patients, we showed virtually all mitochondrial function were inhibited in the heart, suggesting cardiac mitochondrial dysfunction in longer term COVID-19 pathology. In addition, mTOR signaling and the integrated stress response were highly dysregulated throughout all organs. Lastly, mitochondrial inhibition was shown to activate HIF-1α and its target genes shifting cellular metabolism away from catabolism and towards viral synthesis. Our results indicate that manipulation of mitochondrial function may be an important approach for mitigating the severity of COIVID-19.

SARS-CoV-2 infection of human monocytes [55] and human pulmonary alveolar epithelial (HPAEpiC) cells [56] induced mitochondrial reactive oxygen species (mROS) production, increased HIF-1α protein levels and upregulated expression of HIF-1α target genes [57]. The stability of hypoxia inducible factor-1α (HIF-1α) during a SARS-CoV-2 infection was shown to increase the production of pro-inflammatory cytokines and SARS-CoV-2 replication [55–57]. The expression of ORF3a in human embryonic kidney 293 T-antigen cells (HEK293T) cells increased the stability of HIF-1α and induced mROS production, which is an activator of HIF-1α. Together these results suggest that ORF3a induces mROS production to activate HIF-1α, which in turn triggers a shift in cellular metabolism to favor glycolysis, resulting in increased viral replication and transcription of pro-inflammatory cytokines.

Following host cell infection, the SARS-CoV-2 replication/transcription complex synthesizes ~30 kb viral genomes as well as the subgenomic RNAs required to encode for viral structural and mechanistic proteins. Between 1–5 h post-infection, the percentage of coronavirus-encoded protein per total cellular protein translation may increase by as much as 20,000 times, with the fraction of viral to cellular RNA ultimately reaching as high as 90% intracellularly [58]. To accommodate this huge shift towards viral replication, there is certainly a requirement of a shift in cellular metabolism to accommodate for viral synthesis. An investigation of SARS-CoV-2 metabolism during the initial 48-hours post viral infection showed that amino acid availability and synthesis are altered, *de novo* purine synthesis intermediates are accumulated, intracellular glucose and folate are depleted, and lactate levels are elevated [59]. This suggests a viral strategy of upregulating purine metabolism at the post-translational level to coincide with the shutting off of the majority of host proteins at translation levels.

Virus-infected cells also commonly exhibit the Warburg effect - increased glycolytic metabolism in the presence of inadequate oxygen for oxidative phosphorylation - to supply reducing equivalents and precursors for macromolecule biosynthesis, and to support generation of ATP needed for also increasing nucleotide and lipid biosynthesis. Metabolic shifts include dysregulated Ca^2+^ signaling and increased mitochondrial generation of ROS. How SARS-CoV-2 induces host cell nucleotide metabolism remains unanswered.

Mitochondrial metabolism and function are highly impacted in multiple ways. With the shift towards glycolysis, there is a reduction in oxidative phosphorylation affecting the mitochondria and its function. SARS-CoV-2 may interact with the mitochondria to destabilize its oxidative phosphorylation capacity. Coronavirus replication requires the formation of double-membrane vesicles (DMVs) derived from endoplasmic reticulum (ER). These DMVs serve as a site for viral replication and help conceal the virus from host cellular defenses. Interestingly, mitochondrial stress is known to induce mitochondria-derived vesicles (MDVs) that communicate with the ER. It is conceivable that SARS-CoV-2 disruption of mitochondrial function results in the induction of (double-membrane) MDVs. SARS-CoV-2 RNA present in the mitochondria induces mitochondrial dysfunction. Increased DMVs can provide opportunity for viruses to hide and replicate [60].

SARS-CoV-2 and all subgenomic RNAs are enriched in the host mitochondria, and viral genome’s 5ʹ - and 3ʹ -UTRs contain distinct mitochondrial localization signals [61], indicating that the viral RNA may hijack the mitochondria, an interesting hypothesis for experimental validation [61]. Other recent studies have mapped physical interactions of SARS-CoV-2 encoded proteins with mitochondrial localized proteins. These interactions include: NSP8 interaction with mitochondrial ribosomal protein s2 (MRPS2), mitochondrial ribosomal protein s5 (MRPS5), mitochondrial ribosomal protein s25 (MRPS25), and mitochondrial ribosomal protein s27 (MRPS27) ribosomal proteins; ORF9c interaction with mitochondrial NADH:Ubiquinone Oxidoreductase Complex Assembly Factor 1 (NDUFAF1) and NADH:Ubiquinone Oxidoreductase Complex Assembly Factor 9 (NDUFB9); ORF10 interaction with TIMM8; and NSP7 interaction with mitochondrial NADH:Ubiquinone Oxidoreductase Complex Assembly Factor 2 (NDUFAF2). NDUFAF1, NDUFAF2, NDUFB9, and NADH:Ubiquinone Oxidoreductase Complex Assembly Factor 10 (NDUFA10) are all key players in the assembly of complex I, and NDUFA10 is suggested as being one of the master regulators of the SARS-CoV-2 pathology [7]. Interactions were also observed between viral M protein and ATPase Na^+^/K^+^ Transporting Subunit Beta 1 (ATP1B1), ATPase H + Transporting V1 Subunit A (ATP6V1A), acyl-coenzyme A dehydrogenase (ACADM), Alpha-aminoadipic semialdehyde synthase (AASS), Peptidase, Mitochondrial Processing Subunit Beta (PMPCB), Pitrilysin Metallopeptidase 1 (PITRM1), Coenzyme Q8B (COQ8B), and Peptidase, Mitochondrial Processing Subunit Alpha (PMPCA); these proteins are each components of critical mitochondrial metabolic pathways. SARS-CoV-2-encoded ORF9b protein interacts and localizes with Translocase Of Outer Mitochondrial Membrane 70 (TOMM70) [7], a mitochondrial import receptor important for transporting proteins into mitochondria and, more importantly, in modulating anti-viral cellular defense pathways [62]. These mitochondrial interactions offer glimpses of the viral effect on glycolytic and oxidative phosphorylation pathways and the potential side effects.

Another example of COVID-19’s mitochondrial-related impacts is the over-production of cellular ROS [63]. ROS and reactive nitrogen species have diverse functions in biological systems; oxidatively attacking pathogens, regulating cell proliferation, and key signaling functions [64]. However, dysregulation of ROS is implicated in many diseases, including the hyper-inflammatory late phase of COVID-19 [65]. As a part of normal redox metabolism, superoxide radicals are converted into hydrogen peroxide by the action of superoxide dismutase. The hydrogen peroxide is subsequently broken down into water by glutathione peroxidase. During COVID-19, isoforms of enzymes, including glutathione peroxidase and thioredoxin reductase, may be directly targeted and proteolyzed by the SARS-CoV-2 protease, Mpro.

SARS-CoV-2 is thought to suppress the ROS-associated Nuclear factor erythroid 2-related factor 2 (Nrf2) pathway. Nrf2 is a transcription factor that regulates the expression of antioxidant proteins that protect against oxidative damage. Dysregulation of the Nrf2 pathway will exacerbate the pro-oxidative stress caused by the virus [66]. SARS-CoV-2 may suppress the accumulation of the selenoprotein transcripts, which are crucial for the correct functioning of Phospholipid hydroperoxide glutathione peroxidase (GPX4) and mitochondria function [67]. This redox impairment would lead to a buildup of hydrogen peroxide, which could trigger inflammation by promoting the activity of the mitogen-activated protein kinase (MAPK), NF-κB, and the nuclear NOD-like receptor (NLR) family pyrin domain containing 3 (NLRP3) inflammasome [68].

Due to the multiple effects of SARS-CoV-2 that alter cellular metabolic and oxidative states, there are multiple directions to deplete NAD+, loss of cellular ATP and reduced Poly-ADP-ribose polymerase (PARP) activity, each of which have cytotoxic effects of their own [69]. In general, NADPH, synthesized from NAD+, is necessary for many key redox reactions; a reduced level of NADPH could play a mechanistic role in cellular metabolic changes from SARS-CoV-2 infection.

### SARS-CoV-2 mediated reduction in ATP and nitric oxide signaling induces cell stress

**Fig. 4:**
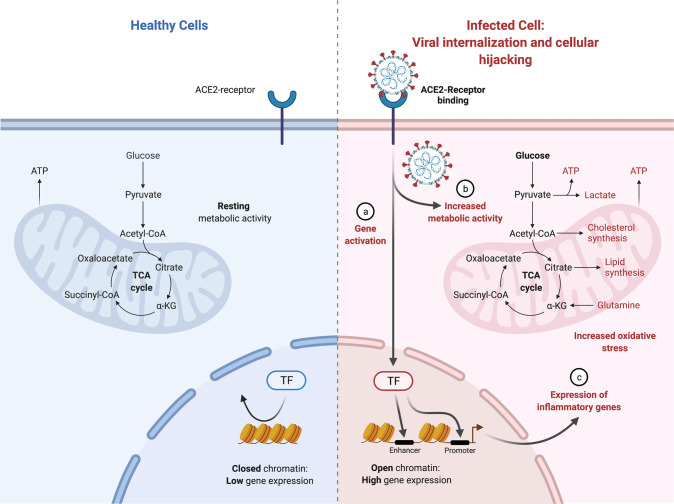
SARS-CoV-2 viral internalization & cellular hijacking. Metabolic pathways and shifts that lead to cellular dysregulation and viral activation to lead towards viral replication (above).

Cellular metabolism adapts to the alterations induced by SARS-CoV-2 infection of the cell (see Fig. 4). These adaptations depend on the cell and tissue type. Here, we focus on ATP signaling, which is relevant to epithelial cells, and nitric oxide (NO) signaling, which tends to be perturbed in endothelial cells [70]. Activation of each pathway at low levels provides protection to the host.

ATP production from oxidative phosphorylation, glycolysis, and other pathways is critical to support cellular physiology, but this molecule also has signaling properties, which can be particularly beneficial in epithelial cells [70]. Perturbations in ATP generation induced by the virus in epithelial cells [71] can lead to ATP release from the apical or basolateral spaces, and subsequent extracellular ATP signaling [72]. It can stimulate P2 receptors on neighboring epithelial cells to activate signal transduction pathways and alter cellular function in adjacent cells even if they are not infected, thus priming naive host cells for confrontation with the virus [71, 72].

In the endothelium, nitric oxide directly affects mitochondrial metabolism through interaction with cytochrome C, providing cytoprotection against free radicals. However, reduction of NO bioavailability, due to the increased oxidative stress state caused by SARS-CoV-2-elevated superoxides, results in the formation of peroxynitrites (ONOO-). The reduced NO diffusion to neighboring vascular smooth muscle may impair vascular function [73]. Peroxynitrite also causes injury to the mitochondria and reduces ATP synthesis, with all of the concomitant negative effects. Therefore, loss of NO bioavailability has major cellular consequences, inducing shifts in multiple enzymatic pathways, cell injury, and death.

Like ATP, NO acts as a biological signaling molecule. This dissolved gas rapidly diffuses across cell membranes and regulates various functions across the body [73]. The vascular endothelium is the predominant cellular source of NO production, and it plays a critical role in maintaining cardiovascular function. Factors that reduce endothelial NO production (increased oxidative stress, changes in NO synthase synthesis) negatively affect endothelial function [73]. The cascade of inflammation and oxidative stress triggered by COVID-19 leads to the formation of superoxide free radicals, impairing biological processes and increasing cytotoxicity in the host cells [74]. The instantaneous reaction of superoxide and NO yields ONOO-, a powerful, cytotoxic nitrating agent. This reaction effectively destroys the NO, rendering it unavailable for its normal regulatory purposes. Thus, the downregulation of NO bioavailability is thought to be a central factor in the severity of COVID-19-associated endotheliitis and the onset of endothelial dysfunction [75].

## Conclusion

The causative agent of the COVID-19 the pandemic, SARS-CoV-2, has caused loss of incomes, economic crises, morbidities, and loss of life worldwide. Here, we describe the virus and review state-of-the-art information about the processes it utilizes to enter and reprogram the human host machinery. We detail research on early infection using evidence from patient samples, organoids and cells, and non-human animal studies. Each of these has limitations but taken together provide unique observational and mechanistic insight on SARS-CoV-2 infection.

COVID-19 is a pleiotropic condition. Viral insults and subsequent cellular metabolic adaptations differ in the context of cell-type, genotype and environmental influences. Thus, much of what we have presented applies to specific cell types and contexts, and we have attempted to cover these contexts.

Key avenues of future research on SARS-CoV-2 infection and propagation include: 1) defining the mechanisms of how the virus enters cells, and the protein and receptor molecules that are critical to this process; 2) elucidating the dynamics of how protein machinery is captured and retrofitted for viral purposes in a cell-specific manner; 3) understanding how the host genetics and environment can affect the ability of the virus to infect; 4) understanding the impact of SARS-CoV-2 on glycolysis and oxidative phosphorylation; and 5) revealing how the mitochondria adapts to ultimately shift its physiology from steady-state.

In the best-case scenario for the SARS-CoV-2 virus, infection leads to a cascade of intracellular adaptations in which multiple networks are remodeled, from transcription to metabolism to signal transduction, shifting the invaded host cell from its original physiology into a SARS-CoV-2 replication system, and causing the emission of new viral particles and signaling molecules. The subsequent disease events will reverberate across the body’s cells and organs. This will be the subject of our Part 2 review (in preparation).

## Supplementary information

Supplemental Table 1

## References

[1] Dong E, Du H, Gardner L (2020). An interactive web-based dashboard to track COVID-19 in real time. Lancet Infect Dis.

[2] Van Egeren D, Novokhodko A, Stoddard M, Tran U, Zetter B, Rogers M (2021). Risk of rapid evolutionary escape from biomedical interventions targeting SARS-CoV-2 spike protein. PloS One.

[3] Yao H, Song Y, Chen Y, Wu N, Xu J, Sun C (2020). Molecular architecture of the SARS-CoV-2 virus. Cell..

[4] Liu J, Xie W, Wang Y, Xiong Y, Chen S, Han J (2020). A comparative overview of COVID-19, MERS and SARS. Int J Surg.

[5] Grifoni A, Sidney J, Zhang Y, Scheuermann RH, Peters B, Sette A (2020). A sequence homology and bioinformatic approach can predict candidate targets for immune responses to SARS-CoV-2. Cell Host Microbe.

[6] Caldaria A, Conforti C, Di Meo N, Dianzani C, Jafferany M, Lotti T, et al. COVID-19 and SARS: Differences and similarities. Dermatol Ther. 2020;33:e13395. 10.1111/dth.13395 PMC7235519 32277530

[7] Gordon DE, Jang GM, Bouhaddou M, Xu J, Obernier K, White KM (2020). A SARS-CoV-2 protein interaction map reveals targets for drug repurposing. Nature..

[8] Benvenuto D, Giovanetti M, Salemi M, Prosperi M, De Flora C, Junior Alcantara LC, Angeletti S, Ciccozzi M (2020). The global spread of 2019-nCoV: a molecular evolutionary analysis. Pathog Glob Health.

[9] Alene M, Yismaw L, Assemie MA, Ketema DB, Mengist B, Kassie B (2021). Magnitude of asymptomatic COVID-19 cases throughout the course of infection: A systematic review and meta-analysis. PloS One.

[10] Mahdy MA, Younis W, Ewaida Z (2020). An overview of SARS-CoV-2 and animal infection. Front Vet Sci.

[11] Fang S, Li K, Shen J, Liu S, Liu J, Yang L (2021). GESS: a database of global evaluation of SARS-CoV-2/hCoV-19 sequences. Nucleic Acids Res.

[12] Banoun H. Evolution of SARS-CoV-2: Review of Mutations, Role of the Host Immune System. Nephron. 2021;145:392–403. 10.1159/000515417 PMC8247830 33910211

[13] Meng B, Kemp SA, Papa G, Datir R, Ferreira IATM, Marelli S, et al. Recurrent emergence of SARS-CoV-2 spike deletion H69/V70 and its role in the Alpha variant B.1.1.7. Cell Rep. 2021;29:109292. 10.1016/j.celrep.2021.109292 PMC8185188 34166617

[14] Centers for Disease Control and Prevention. “SARS-CoV-2 variant classifications and definitions.” (2021).

[15] Letko M, Marzi A, Munster V (2020). Functional assessment of cell entry and receptor usage for SARS-CoV-2 and other lineage B betacoronaviruses. Nat Microbiol.

[16] Dai L, Gao GF (2021). Viral targets for vaccines against COVID-19. Nat Rev Immunol..

[17] Ali A, Vijayan R (2020). Dynamics of the ACE2–SARS-CoV-2/SARS-CoV spike protein interface reveal unique mechanisms. Sci Rep.

[18] Song W, Gui M, Wang X, Xiang Y (2018). Cryo-EM structure of the SARS coronavirus spike glycoprotein in complex with its host cell receptor ACE2. PLoS Pathog.

[19] Chu H, Chan JF, Yuen TT, Shuai H, Yuan S, Wang Y (2020). Comparative tropism, replication kinetics, and cell damage profiling of SARS-CoV-2 and SARS-CoV with implications for clinical manifestations, transmissibility, and laboratory studies of COVID-19: an observational study. Lancet Microbe.

[20] Yan R, Zhang Y, Li Y, Ye F, Guo Y, Xia L (2021). Structural basis for the different states of the spike protein of SARS-CoV-2 in complex with ACE2. Cell Res.

[21] Puelles VG, Lütgehetmann M, Lindenmeyer MT, Sperhake JP, Wong MN, Allweiss L (2020). Multiorgan and renal tropism of SARS-CoV-2. N. Engl J Med.

[22] Menter T, Haslbauer JD, Nienhold R, Savic S, Hopfer H, Deigendesch N (2020). Postmortem examination of COVID‐19 patients reveals diffuse alveolar damage with severe capillary congestion and variegated findings in lungs and other organs suggesting vascular dysfunction. Histopathology..

[23] Ziegler CG, Allon SJ, Nyquist SK, Mbano IM, Miao VN, Tzouanas CN (2020). SARS-CoV-2 receptor ACE2 is an interferon-stimulated gene in human airway epithelial cells and is detected in specific cell subsets across tissues. Cell..

[24] Park J, Foox J, Hether T, Danko DC, Warren S, Kim Y, et al. System-wide transcriptome damage and tissue identity loss in COVID-19 patients. Cell Rep Med. 2022;3:100522. 10.1016/j.xcrm.2022.100522 PMC8784611 35233546

[25] Han Y, Duan X, Yang L, Nilsson-Payant BE, Wang P, Duan F (2021). Identification of SARS-CoV-2 inhibitors using lung and colonic organoids. Nature..

[26] Ashraf UM, Abokor AA, Edwards JM, Waigi EW, Royfman RS, Hasan SA (2021). SARS-CoV-2, ACE2 expression, and systemic organ invasion. Physiological Genomics.

[27] Suryamohan K, Diwanji D, Stawiski EW, Gupta R, Miersch S, Liu J (2021). Human ACE2 receptor polymorphisms and altered susceptibility to SARS-CoV-2. Commun Biol.

[28] Ozono S, Zhang Y, Ode H, Sano K, Tan TS, Imai K (2021). SARS-CoV-2 D614G spike mutation increases entry efficiency with enhanced ACE2-binding affinity. Nat Commun.

[29] Sodhi CP, Wohlford-Lenane C, Yamaguchi Y, Prindle T, Fulton WB, Wang S (2018). Attenuation of pulmonary ACE2 activity impairs inactivation of des-Arg9 bradykinin/BKB1R axis and facilitates LPS-induced neutrophil infiltration. Am J Physiol-Lung Cell Mol Physiol.

[30] Calixto JB, Medeiros R, Fernandes ES, Ferreira J, Cabrini DA, Campos MM (2004). Kinin B1 receptors: key G‐protein‐coupled receptors and their role in inflammatory and painful processes. Br J Pharmacol.

[31] Huang Y, Yang C, Xu XF, Xu W, Liu SW (2020). Structural and functional properties of SARS-CoV-2 spike protein: potential antivirus drug development for COVID-19. Acta Pharmacologica Sin.

[32] Lan J, Ge J, Yu J, Shan S, Zhou H, Fan S (2020). Structure of the SARS-CoV-2 spike receptor-binding domain bound to the ACE2 receptor. Nature..

[33] Davidson AM, Wysocki J, Batlle D (2020). Interaction of SARS-CoV-2 and other coronavirus with ACE (angiotensin-converting enzyme)-2 as their main receptor: therapeutic implications. Hypertension.

[34] Ko PJ, Woodrow C, Dubreuil MM, Martin BR, Skouta R, Bassik MC (2019). A ZDHHC5-GOLGA7 protein acyltransferase complex promotes nonapoptotic cell death. Cell Chem Biol.

[35] Van De Veerdonk F, Netea MG, Van Deuren M, Van Der Meer JW, De Mast Q, Bruggemann RJ, et al. Kinins and cytokines in COVID-19: a comprehensive pathophysiological approach.

[36] Mohammad S, Bouchama A, Mohammad Alharbi B, Rashid M, Saleem Khatlani T, Gaber NS (2020). SARS-CoV-2 ORF8 and SARS-CoV ORF8ab: genomic divergence and functional convergence. Pathogens..

[37] Castaño-Rodriguez C, Honrubia JM, Gutiérrez-Álvarez J, DeDiego ML, Nieto-Torres JL, Jimenez-Guardeño JM (2018). Role of severe acute respiratory syndrome coronavirus viroporins E, 3a, and 8a in replication and pathogenesis. MBio.

[38] McClenaghan C, Hanson A, Lee SJ, Nichols CG (2020). Coronavirus proteins as ion channels: Current and potential research. Front Immunol.

[39] Di Filippo L, Formenti AM, Rovere-Querini P, Carlucci M, Conte C, Ciceri F (2020). Hypocalcemia is highly prevalent and predicts hospitalization in patients with COVID-19. Endocrine..

[40] Schett G, Manger B, Simon D, Caporali R COVID-19 revisiting inflammatory pathways of arthritis. Nat Rev Rheumatol. 2020;16:465–70. 10.1038/s41584-020-0451-z PMC7304381 32561873

[41] Huang J, Hume AJ, Abo KM, Werder RB, Villacorta-Martin C, Alysandratos KD (2020). SARS-CoV-2 infection of pluripotent stem cell-derived human lung alveolar type 2 cells elicits a rapid epithelial-intrinsic inflammatory response. Cell Stem Cell.

[42] Zheng M, Karki R, Williams EP, Yang D, Fitzpatrick E, Vogel P, et al. TLR2 senses the SARS-CoV-2 envelope protein to produce inflammatory cytokines. Nat Immunology. 2021;1-0. 10.1038/s41590-021-00937-x PMC8882317 33963333

[43] Mukherjee R, Bhattacharya A, Bojkova D, Mehdipour AR, Shin D, Khan KS, et al. Famotidine inhibits toll-like receptor 3-mediated inflammatory signaling in SARS-CoV-2 infection. J Biolog Chem. 2021;297:100925. 10.1016/j.jbc.2021.100925 PMC8241579 34214498

[44] Zhang Q, Bastard P, Liu Z, Le Pen J, Moncada-Velez M, Chen J, et al. Inborn errors of type I IFN immunity in patients with life-threatening COVID-19. Science. 2020;370:eabd4570. 10.1126/science.abd4570 PMC7857407 32972995

[45] Bastard P, Rosen LB, Zhang Q, Michailidis E, Hoffmann HH, Zhang Y, et al. Autoantibodies against type I IFNs in patients with life-threatening COVID-19. Science. 2020;370:eabd4585. 10.1126/science.abd4585 PMC7857397 32972996

[46] Hung IF, Lung KC, Tso EY, Liu R, Chung TW, Chu MY (2020). Triple combination of interferon beta-1b, lopinavir–ritonavir, and ribavirin in the treatment of patients admitted to hospital with COVID-19: an open-label, randomised, phase 2 trial. Lancet.

[47] Irvani SS, Golmohammadi M, Pourhoseingholi MA, Shokouhi S, Darazam IA (2020). Effectiveness of Interferon Beta 1a, compared to Interferon Beta 1b and the usual therapeutic regimen to treat adults with moderate to severe COVID-19: structured summary of a study protocol for a randomized controlled trial. Trials..

[48] Thevarajan I, Buising KL, Cowie BC (2020). Clinical presentation and management of COVID-19. Med J Aust.

[49] Tan L, Wang Q, Zhang D, Ding J, Huang Q, Tang YQ (2020). Lymphopenia predicts disease severity of COVID-19: a descriptive and predictive study. Signal Transduct Target Ther.

[50] Yang J, Wise L, Fukuchi KI (2020). TLR4 Cross-talk with NLRP3 inflammasome and complement signaling pathways in Alzheimer’s disease. Front Immunol.

[51] Li JY, Liao CH, Wang Q, Tan YJ, Luo R, Qiu Y (2020). The ORF6, ORF8 and nucleocapsid proteins of SARS-CoV-2 inhibit type I interferon signaling pathway. Virus Res.

[52] Lei X, Dong X, Ma R, Wang W, Xiao X, Tian Z (2020). Activation and evasion of type I interferon responses by SARS-CoV-2. Nat Commun.

[53] Codo AC, Davanzo GG, de Brito Monteiro L, de Souza GF, Muraro SP, Virgilio-da-Silva JV (2020). Elevated glucose levels favor SARS-CoV-2 infection and monocyte response through a HIF-1α/glycolysis-dependent axis. Cell Metab.

[54] Guarnieri JW, Dybas JM, Fazelinia H, Kim MS, Frere J, Zhang Y, et al. Targeted down regulation of core mitochondrial genes during SARS-CoV-2 infection. bioRxiv. 2022 Feb 22.

[55] Wang P, Luo R, Zhang M, Wang Y, Song T, Tao T (2020). A cross-talk between epithelium and endothelium mediates human alveolar–capillary injury during SARS-CoV-2 infection. Cell death Dis.

[56] Tian M, Liu W, Li X, Zhao P, Shereen MA, Zhu C (2021). HIF-1α promotes SARS-CoV-2 infection and aggravates inflammatory responses to COVID-19. Signal Transduct Target Ther.

[57] Irigoyen N, Firth AE, Jones JD, Chung BY, Siddell SG, Brierley I (2016). High-resolution analysis of coronavirus gene expression by RNA sequencing and ribosome profiling. PLoS Pathog.

[58] Singh KK, Chaubey G, Chen JY, Suravajhala P (2020). Decoding SARS-CoV-2 hijacking of host mitochondria in COVID-19 pathogenesis. Am J Physiol-Cell Physiol.

[59] Ajaz S, McPhail MJ, Singh KK, Mujib S, Trovato FM, Napoli S (2021). Mitochondrial metabolic manipulation by SARS-CoV-2 in peripheral blood mononuclear cells of patients with COVID-19. Am J Physiol-Cell Physiol.

[60] Stukalov A, Girault V, Grass V, Karayel O, Bergant V, Urban C (2021). Multilevel proteomics reveals host perturbations by SARS-CoV-2 and SARS-CoV. Nature.

[61] Thaker SK, Ch’ng J, Christofk HR (2019). Viral hijacking of cellular metabolism. BMC Biol.

[62] Cecchini R, Cecchini AL (2020). SARS-CoV-2 infection pathogenesis is related to oxidative stress as a response to aggression. Med Hypotheses.

[63] Suhail S, Zajac J, Fossum C, Lowater H, McCracken C, Severson N, et al. Role of oxidative stress on SARS-CoV (SARS) and SARS-CoV-2 (COVID-19) infection: a review. The protein journal. 2020;39:644–56. 10.1007/s10930-020-09935-8 PMC7587547 33106987

[64] Taylor EW, Radding W (2020). Understanding selenium and glutathione as antiviral factors in COVID-19: Does the viral Mpro protease target host selenoproteins and glutathione synthesis?. Front Nutr.

[65] Wang Y, Huang J, Sun Y, Stubbs D, He J, Li W (2021). SARS-CoV-2 suppresses mRNA expression of selenoproteins associated with ferroptosis, endoplasmic reticulum stress and DNA synthesis. Food Chem Toxicol.

[66] Abais JM, Xia M, Zhang Y, Boini KM, Li PL (2015). Redox regulation of NLRP3 inflammasomes: ROS as trigger or effector?. Antioxid Redox Signal.

[67] Olagnier D, Farahani E, Thyrsted J, Blay-Cadanet J, Herengt A, Idorn M (2020). SARS-CoV2-mediated suppression of NRF2-signaling reveals potent antiviral and anti-inflammatory activity of 4-octyl-itaconate and dimethyl fumarate. Nat Commun.

[68] Heer CD, Sanderson DJ, Voth LS, Alhammad YM, Schmidt MS, Trammell SA (2020). Coronavirus infection and PARP expression dysregulate the NAD metabolome: An actionable component of innate immunity. J Biol Chem.

[69] Agledal L, Niere M, Ziegler M (2010). The phosphate makes a difference: cellular functions of NADP. Redox Rep.

[70] Schwiebert EM, Zsembery A (2003). Extracellular ATP as a signaling molecule for epithelial cells. Biochimica et Biophysica Acta (BBA)-Biomembranes.

[71] Novak I (2003). ATP as a signaling molecule: the exocrine focus. Physiology..

[72] Green SJ (2020). Covid-19 accelerates endothelial dysfunction and nitric oxide deficiency. Microbes Infect.

[73] Yamasaki H (2020). Blood nitrate and nitrite modulating nitric oxide bioavailability: potential therapeutic functions in COVID-19. Nitric Oxide.

[74] Lingappan K (2018). NF-κB in oxidative stress. Curr Opin Toxicol.

[75] Ozdemir B, Yazici A (2020). Could the decrease in the endothelial nitric oxide (NO) production and NO bioavailability be the crucial cause of COVID-19 related deaths?. Med Hypotheses.

