# Supplementary material for: A comprehensive SARS-CoV-2 and COVID-19 review, Part 1: Intracellular overdrive for SARS-CoV-2 infection
Source: Eur J Hum Genet. 2022 May 16;30(8):889–98. doi: 10.1038/s41431-022-01108-8 (PMC9108708; doi:10.1038/s41431-022-01108-8)
Supplement: Supplementary file 1 — Supplemental Table 1 [file 41431_2022_1108_MOESM1_ESM.pdf]

Supplementary Table 1: List of viral components and functions

	Main Function(s)	Molecular Weight (Kd)	Homolgy to CoV1 (vs. Wuhan)	Homolgy to 1.612.2 (vs. Wuhan)	REF
**NSP1**	Cellular mRNA degradation, global translation inhibition, disrupts splicing	19.78	84.44	100	(1, 2, 3, 4)
**NSP2**	Disrupts cell cycle progression	70.52	68.34	100	
**NSP3**	Formation of double-membrane vesicles (DMVs), SARS-CoV protease, Type I IFN antagonist	217.28	75.97	99.18	(4, 5, 6, 7, 8, 9)
**NSP4**	Formation of DMVs	56.19	80	99.6	(8, 9)
**NSP5**	Main SARS-CoV protease	33.8	96.08	100	(10)
**NSP6**	Formation of DMVs, Type I IFN antagonist	33.04	88.15	99.66	(9, 11)
**NSP7**	Replication complex, Interferes with protein trafficking	9.24	98.8	100	(12, 13, 14)
**NSP8**	Primase, Interferes with protein trafficking	21.89	97.47	100	(3, 12, 13, 14)
**NSP9**	RNA binding protein, Interferes with protein trafficking	12.38	97.35	100	(3, 15, 16)
**NSP10**	Cofactor of NSP 14 & 16	14.79	97.12	100	(17, 18)
**NSP11**	Unknown	1.33	84.62	100	
**NSP12**	RNA-dependent RNA polymerase, Type I IFN antagonist	106.76	96.14	99.79	(4, 12, 19, 20)
**NSP13**	RNA helicase, 5' phosphatase, Type I IFN antagonist	66.86	99.83	99.83	(4, 11, 21, 22)
**NSP14**	N7-MTase, 3'-5' exonuclease, Type I IFN antagonist	59.82	95.07	99.81	(4, 17, 23, 24, 25)
**NSP15**	Endonuclease	38.82	88.73	100	(26)
**NSP16**	2'-O-MTase, mRNA capping, Inhihits global mRNA splicing	33.33	93.29	100	(3, 17, 27, 28)
**S**	Binds the host cell receptor (hACE2)	141.2	75.96	99.14	(29, 30, 31)
**M**	Membrane, Type I IFN antagonist	25.15	90.54	99.55	(4, 11, 30, 31, 33)
**E**	Envelope protein, Involved in viral morphogenesis and assembly, Activates the NLRP3-inflammasome, Viroporin	8.37	94.74	100	(30, 31, 33, 34, 35, 36)

**N**	Nucleocapsid phosphoprotein, binds to RNA genome	45.64	90.52	99.05	(36, 37, 38)
**ORF3a**	Activates the NLRP3-inflammasome, Type I IFN antagonist, Viroproin	31.13	72.36	99.64	(4, 11, 39, 40)
**ORF6**	Type I IFN antagonist	7.27	68.85	100	(4, 11)
**ORF7a**	Type I IFN antagonist	13.75	85.25	99.17	(11)
**ORF7b**	Type I IFN antagonist	5.18	85.37	100	(11)
**ORF8**	Type I IFN antagonist, Pro-inflammatory cytokine agonist	13.83	10.74	100	(41, 42)
**ORF9b**	Interacts with TOMM70, Inhibits MAVS antiviral signaling, Type I IFN antagonist	10.8	72.42	100	(43, 44)
**ORF10**	Unknown	4.45	N/A	100	

Supplemental References:

1. Thoms M, Buschauer R, Ameismeier M, Koepke L, Denk T, Hirschenberger M, Kratzat H, Hayn M, Mackens-Kiani T, Cheng J, Straub JH. Structural basis for translational shutdown and immune evasion by the Nsp1 protein of SARS-CoV-2. Science. 2020 Sep 4;369(6508):1249-55.
2. Lapointe CP, Grosely R, Johnson AG, Wang J, Fernández IS, Puglisi JD. Dynamic competition between SARS-CoV-2 NSP1 and mRNA on the human ribosome inhibits translation initiation. Proceedings of the National Academy of Sciences. 2021 Feb 9;118(6).
3. Banerjee AK, Blanco MR, Bruce EA, Honson DD, Chen LM, Chow A, Bhat P, Ollikainen N, Quinodoz SA, Loney C, Thai J. SARS-CoV-2 disrupts splicing, translation, and protein trafficking to suppress host defenses. Cell. 2020 Nov 25;183(5):1325-39.
4. Lei X, Dong X, Ma R, Wang W, Xiao X, Tian Z, Wang C, Wang Y, Li L, Ren L, Guo F. Activation and evasion of type I interferon responses by SARS-CoV-2. Nature communications. 2020 Jul 30;11(1):1-2.
5. Liu G, Lee JH, Parker ZM, Acharya D, Chiang JJ, van Gent M, Riedl W, Davis-Gardner ME, Wies E, Chiang C, Gack MU. ISG15-dependent activation of the sensor MDA5 is antagonized by the SARS-CoV-2 papain-like protease to evade host innate immunity. Nature Microbiology. 2021 Apr;6(4):467-78.
6. Wang L, Hu W, Fan C. Structural and biochemical characterization of SADS-CoV papain-like protease 2. Protein Science. 2020 May;29(5):1228-41.
7. Armstrong LA, Lange SM, Dee Cesare V, Matthews SP, Nirujogi RS, Cole I, Hope A, Cunningham F, Toth R, Mukherjee R, Bojkova D. Biochemical characterization of protease activity of Nsp3 from SARS-CoV-2 and its inhibition by nanobodies. PloS one. 2021 Jul 16;16(7):e0253364.
8. Hagemeijer MC, Monastyrska I, Griffith J, van der Sluijs P, Voortman J, en Henegouwen PM, Vonk AM, Rottier PJ, Reggiori F, De Haan CA. Membrane rearrangements mediated by coronavirus nonstructural proteins 3 and 4. Virology. 2014 Jun 1;458:125-35.
9. Angelini MM, Akhlaghpour M, Neuman BW, Buchmeier MJ. Severe acute respiratory syndrome coronavirus nonstructural proteins 3, 4, and 6 induce double-membrane vesicles. MBio. 2013 Aug 13;4(4):e00524-13.
10. Lee J, Worrall LJ, Vuckovic M, Rosell FI, Gentile F, Ton AT, Caveney NA, Ban F, Cherkasov A, Paetzel M, Strynadka NC. Crystallographic structure of wild-type SARS-CoV-2 main protease acyl-enzyme intermediate with physiological C-terminal autoprocessing site. Nature communications. 2020 Nov 18;11(1):1-9.
11. Xia H, Cao Z, Xie X, Zhang X, Chen JY, Wang H, Menachery VD, Rajsbaum R, Shi PY. Evasion of type I interferon by SARS-CoV-2. Cell reports. 2020 Oct 6;33(1):108234.
12. Hillen HS, Kokic G, Farnung L, Dienemann C, Tegunov D, Cramer P. Structure of replicating SARS-CoV-2 polymerase. Nature. 2020 Aug;584(7819):154-6.
13. Peng Q, Peng R, Yuan B, Zhao J, Wang M, Wang X, Wang Q, Sun Y, Fan Z, Qi J, Gao GF. Structural and biochemical characterization of the nsp12-nsp7-nsp8

core polymerase complex from SARS-CoV-2. Cell reports. 2020 Jun 16;31(11):107774.
14. Yan L, Zhang Y, Ge J, Zheng L, Gao Y, Wang T, Jia Z, Wang H, Huang Y, Li M, Wang Q. Architecture of a SARS-CoV-2 mini replication and transcription complex. Nature communications. 2020 Nov 18;11(1):1-6.
15. Littler DR, Gully BS, Colson RN, Rossjohn J. Crystal structure of the SARS-CoV-2 non-structural protein 9, Nsp9. Iscience. 2020 Jul 24;23(7):101258.
16. El-Kamand S, Du Plessis MD, Breen N, Johnson L, Beard S, Kwan AH, Richard DJ, Cubeddu L, Gamsjaeger R. A distinct ssDNA/RNA binding interface in the Nsp9 protein from SARS-CoV-2. Proteins: Structure, Function, and Bioinformatics. 2021 Aug 8.
17. Wilamowski M, Sherrell DA, Minasov G, Kim Y, Shuvalova L, Lavens A, Chard R, Maltseva N, Jedrzejczak R, Rosas-Lemus M, Saint N. 2′-O methylation of RNA cap in SARS-CoV-2 captured by serial crystallography. Proceedings of the National Academy of Sciences. 2021 May 25;118(21).
18. Viswanathan T, Misra A, Chan SH, Qi S, Dai N, Arya S, Martinez-Sobrido L, Gupta YK. A metal ion orients SARS-CoV-2 mRNA to ensure accurate 2′-O methylation of its first nucleotide. Nature communications. 2021 Jun 2;12(1):1-7.
19. Wang W, Zhou Z, Xiao X, Tian Z, Dong X, Wang C, Li L, Ren L, Lei X, Xiang Z, Wang J. SARS-CoV-2 nsp12 attenuates type I interferon production by inhibiting IRF3 nuclear translocation. Cellular & Molecular Immunology. 2021 Apr;18(4):945-53.
20. Gao Y, Yan L, Huang Y, Liu F, Zhao Y, Cao L, Wang T, Sun Q, Ming Z, Zhang L, Ge J. Structure of the RNA-dependent RNA polymerase from COVID-19 virus. Science. 2020 May 15;368(6492):779-82.
21. Chen J, Malone B, Llewellyn E, Grasso M, Shelton PM, Olinares PD, Maruthi K, Eng ET, Vatandaslar H, Chait BT, Kapoor TM. Structural basis for helicase-polymerase coupling in the SARS-CoV-2 replication-transcription complex. Cell. 2020 Sep 17;182(6):1560-73.
22. Newman JA, Douangamath A, Yazdani S, Yosaatmadja Y, Aimon A, Brandao-Neto J, Dunnett L, Gorrie-Stone T, Skyner R, Fearon D, Schapira M. Structure, Mechanism and Crystallographic fragment screening of the SARS-CoV-2 NSP13 helicase. bioRxiv. 2021 Jan 1.
23. Hsu JC, Laurent-Rolle M, Pawlak JB, Wilen CB, Cresswell P. Translational shutdown and evasion of the innate immune response by SARS-CoV-2 NSP14 protein. Proceedings of the National Academy of Sciences. 2021 Jun 15;118(24).
24. Yan L, Yang Y, Li M, Zhang Y, Zheng L, Ge J, Huang YC, Liu Z, Wang T, Gao S, Zhang R. Coupling of N7-methyltransferase and 3′-5′ exoribonuclease with SARS-CoV-2 polymerase reveals mechanisms for capping and proofreading. Cell. 2021 Jun 24;184(13):3474-85.
25. Lin S, Chen H, Chen Z, Yang F, Ye F, Zheng Y, Yang J, Lin X, Sun H, Wang L, Wen A. Crystal structure of SARS-CoV-2 nsp10 bound to nsp14-ExoN domain reveals an exoribonuclease with both structural and functional integrity. Nucleic acids research. 2021 May 21;49(9):5382-92.
26. Pillon MC, Frazier MN, Dillard LB, Williams JG, Kocaman S, Krahn JM, Perera L, Hayne CK, Gordon J, Stewart ZD, Sobhany M. Cryo-EM structures of the SARS-

CoV-2 endoribonuclease Nsp15 reveal insight into nuclease specificity and dynamics. Nature communications. 2021 Jan 27;12(1):1-2.
27. Minasov G, Rosas-Lemus M, Shuvalova L, Inniss NL, Brunzelle JS, Daczkowski CM, Hoover P, Mesecar AD, Satchell KJ. Mn2+ coordinates Cap-0-RNA to align substrates for efficient 2′-O-methyl transfer by SARS-CoV-2 nsp16. Science Signaling. 2021 Jun 15.
28. Viswanathan T, Arya S, Chan SH, Qi S, Dai N, Misra A, Park JG, Oladunni F, Kovalskyy D, Hromas RA, Martinez-Sobrido L. Structural basis of RNA cap modification by SARS-CoV-2. Nature communications. 2020 Jul 24;11(1):1-7.
29. Huang Y, Yang C, Xu XF, Xu W, Liu SW. Structural and functional properties of SARS-CoV-2 spike protein: potential antivirus drug development for COVID-19. Acta Pharmacologica Sinica. 2020 Sep;41(9):1141-9.
30. Swann H, Sharma A, Preece B, Peterson A, Eldredge C, Belnap DM, Vershinin M, Saffarian S. Minimal system for assembly of SARS-CoV-2 virus like particles. Scientific reports. 2020 Dec 14;10(1):1-5.
31. Boson B, Legros V, Zhou B, Siret E, Mathieu C, Cosset FL, Lavillette D, Denolly S. The SARS-CoV-2 envelope and membrane proteins modulate maturation and retention of the spike protein, allowing assembly of virus-like particles. Journal of Biological Chemistry. 2021 Jan 1;296.
32. Fu YZ, Wang SY, Zheng ZQ, Huang Y, Li WW, Xu ZS, Wang YY. SARS-CoV-2 membrane glycoprotein M antagonizes the MAVS-mediated innate antiviral response. Cellular & Molecular Immunology. 2021 Mar;18(3):613-20.
33. Nieto-Torres JL, DeDiego ML, Verdiá-Báguena C, Jimenez-Guardeño JM, Regla-Nava JA, Fernandez-Delgado R, Castaño-Rodriguez C, Alcaraz A, Torres J, Aguilella VM, Enjuanes L. Severe acute respiratory syndrome coronavirus envelope protein ion channel activity promotes virus fitness and pathogenesis. PLoS pathogens. 2014 May 1;10(5):e1004077.
34. Mandala VS, McKay MJ, Shcherbakov AA, Dregni AJ, Kolocouris A, Hong M. Structure and drug binding of the SARS-CoV-2 envelope protein transmembrane domain in lipid bilayers. Nature structural & molecular biology. 2020 Dec;27(12):1202-8.
35. Xia B, Shen X, He Y, Pan X, Liu FL, Wang Y, Yang F, Fang S, Wu Y, Duan Z, Zuo X. SARS-CoV-2 envelope protein causes acute respiratory distress syndrome (ARDS)-like pathological damages and constitutes an antiviral target. Cell research. 2021 Jun 10:1-4.
36. Carlson CR, Asfaha JB, Ghent CM, Howard CJ, Hartooni N, Safari M, Frankel AD, Morgan DO. Phosphoregulation of phase separation by the SARS-CoV-2 N protein suggests a biophysical basis for its dual functions. Molecular cell. 2020 Dec 17;80(6):1092-103.
37. Dinesh DC, Chalupska D, Silhan J, Koutna E, Nencka R, Veverka V, Boura E. Structural basis of RNA recognition by the SARS-CoV-2 nucleocapsid phosphoprotein. PLoS pathogens. 2020 Dec 2;16(12):e1009100.
38. Iserman C, Roden CA, Boerneke MA, Sealfon RS, McLaughlin GA, Jungreis I, Fritch EJ, Hou YJ, Ekena J, Weidmann CA, Theesfeld CL. Genomic RNA elements drive phase separation of the SARS-CoV-2 nucleocapsid. Molecular cell. 2020 Dec 17;80(6):1078-91.

39. Kern DM, Sorum B, Mali SS, Hoel CM, Sridharan S, Remis JP, Toso DB, Kotecha A, Bautista DM, Brohawn SG. Cryo-EM structure of SARS-CoV-2 ORF3a in lipid nanodiscs. Nature Structural & Molecular Biology. 2021 Jun 22:1-0.
40. Chen IY, Moriyama M, Chang MF, Ichinohe T. Severe acute respiratory syndrome coronavirus viroporin 3a activates the NLRP3 inflammasome. Frontiers in microbiology. 2019 Jan 29;10:50.
41. Lin X, Fu B, Yin S, Li Z, Liu H, Zhang H, Xing N, Wang Y, Xue W, Xiong Y, Zhang S. ORF8 contributes to cytokine storm during SARS-CoV-2 infection by activating IL-17 pathway. Iscience. 2021 Apr 23;24(4):102293.
42. Li JY, Liao CH, Wang Q, Tan YJ, Luo R, Qiu Y, Ge XY. The ORF6, ORF8 and nucleocapsid proteins of SARS-CoV-2 inhibit type I interferon signaling pathway. Virus research. 2020 Sep 1;286:198074.
43. Wu J, Shi Y, Pan X, Wu S, Hou R, Zhang Y, Zhong T, Tang H, Du W, Wang L, Wo J. SARS-CoV-2 ORF9b inhibits RIG-I-MAVS antiviral signaling by interrupting K63-linked ubiquitination of NEMO. Cell reports. 2021 Feb 16;34(7):108761.
44. Jiang HW, Zhang HN, Meng QF, Xie J, Li Y, Chen H, Zheng YX, Wang XN, Qi H, Zhang J, Wang PH. SARS-CoV-2 Orf9b suppresses type I interferon responses by targeting TOM70. Cellular & molecular immunology. 2020 Sep;17(9):998-1000.
